# Risk factors for low back pain in active military personnel: a systematic review

**DOI:** 10.1186/s12998-021-00409-x

**Published:** 2021-12-30

**Authors:** Daphne To, Mana Rezai, Kent Murnaghan, Carol Cancelliere

**Affiliations:** 1 Canadian Memorial Chiropractic College, Toronto, ON Canada; 2 Institute for Disability and Rehabilitation Research, Ontario Tech University and Canadian Memorial Chiropractic College, Oshawa, ON Canada; 3 Faculty of Health Sciences, Ontario Tech University, Oshawa, ON Canada

**Keywords:** Low back pain, Risk factor, Military, Systematic review

## Abstract

**Purpose:**

Low back pain (LBP) is prevalent in military personnel. We aimed to systematically review the literature regarding risk factors for first-time LBP during military service among active duty military personnel.

**Methods:**

We searched six electronic databases (inception-April 2020) for randomised controlled trials, cohort studies, and case–control studies published in English in peer-reviewed journals. Eligible studies were independently critically appraised by paired reviewers and a descriptive synthesis was conducted.

**Results:**

We screened 1981 records, reviewed 118 full-text articles, and synthesised data from eight acceptable quality cohort studies. Studies assessed physical (n = 4), sociodemographic (n = 2), and/or occupational factors (n = 5) associated with LBP. Two studies reported prior LBP was associated with a greater than twofold increased risk of LBP compared to those without prior LBP. Other factors consistently associated with LBP included previous musculoskeletal injury (n = 2), less time spent on physical training (n = 2), female sex (n = 2), and lower rank (n = 2). Factors associated with LBP from single studies included marital status, lower education level, blast injury, job duties, and service type. We found inconsistent associations for performance on physical fitness tests, age, and occupation type. Psychological risk factors were not assessed in any included studies.

**Conclusion:**

In active duty personnel, prior history of LBP, previous musculoskeletal injury, less time in physical training, female sex, and lower rank were consistent risk factors for LBP. This information is relevant for researchers, active duty military personnel, and other decision makers. Future studies should explore causal relationships for LBP in this population.

* PROSPERO registration number*: CRD42018084549.

**Supplementary Information:**

The online version contains supplementary material available at 10.1186/s12998-021-00409-x.

## Introduction

Musculoskeletal (MSK) disorders, particularly low back pain (LBP), are highly prevalent and are one of the leading causes of disability in the general population [1, 2]. Similarly, the prevalence of LBP in military personnel is also high [3, 4]. An analysis of the United States (U.S.) Navy and Marine Corps Physical Evaluation Boards over a 1-year period demonstrated that MSK disorders were the most frequently diagnosed condition (43%), with back pain being the most frequently identified MSK disorder (29%) [5]. In the U.S. active duty military population, the overall incidence rate of LBP was 40.5 per 1000 person-years over an 8-year period [6]. However, the true burden of MSK disorders and LBP in this population may actually be higher, as there may be an underreporting of MSK injuries due to the fear of affecting future career opportunities [7]. Musculoskeletal disorders are a substantial financial burden [8], a common reason for medical evacuation during military duty, and reduce the probability of return to duty [9].

A variety of risk factors for LBP in the general population have been identified from previous systematic reviews [10, 11]. Specifically for workers, some occupational demands such as heavy lifting, awkward postures, and bending may be risk factors for LBP, although a causal relationship has not yet been determined [12–14]. While these reviews have looked at risk factors for LBP in various occupational settings, to our knowledge, no reviews have looked specifically at risk factors for LBP in the active duty military population. Given that the burden of LBP is high in this population and that their daily tasks are both physically and psychologically demanding, there is a need to better understand factors that may contribute to the development of LBP in this population. By understanding risk factors for LBP, prevention strategies may be developed and targeted to reduce the burden of LBP in active duty military personnel.

Therefore, the objective of this study was to critically appraise and synthesise the literature examining risk factors of incident LBP in active duty military personnel.

## Methods

### Study design

The systematic review was conducted and reported according to the Preferred Reporting Items for Systematic Reviews and Meta-Analyses (PRISMA) statement (Additional file 1) [15]. The protocol was registered with the International Prospective Register of Systematic Reviews (PROSPERO) [16] on January 16, 2018 and updated on August 28, 2020 (registration no. CRD42018084549).

### Search strategy

The search strategy was developed in consultation with a health sciences librarian (KM) and reviewed by a second librarian using the Peer Review of Electronic Search Strategies (PRESS) Checklist [17]. The electronic databases PubMed, MEDLINE (EBSCO), Cumulative Index to Nursing and Allied Health Literature (EBSCO), Cochrane Database for Registered Trials, PsycINFO (OVID), and Embase (OVID) were systematically searched from database inception to March 2, 2018 and updated on April 15, 2020. The reference lists of all eligible articles were hand-searched to identify additional articles. Search terms consisted of subject headings specific to each database (e.g., MeSH in MEDLINE) and free text words relevant to military personnel, LBP, and risk factors (Additional file 2).

### Eligibility criteria

#### Study population

Active military personnel were defined as individuals 16 years of age and older who were in active military duty at the time of the study. This population included members of the Armed Forces, Navy, and Air Force. Study populations of retired military personnel or those with a history of previous LBP during military service were excluded.

#### Risk factors

We searched for all risk factors including risk markers, predictors, and risk determinants. Risk markers are factors that are associated with an outcome of interest; predictors result from prediction model studies and may either be causal or non-causal; risk determinants are a cause of the outcome (causal modelling) [18]. We searched for risk factors in any domain, such as sociodemographic, physical, psychological, or occupational risk factors.

#### Outcomes

Low back pain was defined according to the European Guidelines for Prevention in Low Back Pain [19] as pain and discomfort, localised below the costal margin and above the inferior gluteal folds, with or without leg pain. Low back pain as a result of fracture/dislocation, infection, cancer, or other serious low back pathology were excluded. We included only individuals with incident LBP—defined as a new episode or first occurrence of LBP during military service. There was no minimum follow up period required for outcome assessment.

#### Study design

Randomised controlled trials, cohort studies, and case–control studies were included. We excluded cross-sectional studies, pilot studies, case reports or series, biomechanical studies, laboratory studies, qualitative studies, reviews (i.e., systematic reviews, meta-analyses, and narrative reviews), and guidelines.

#### Publication type

Articles published in English in peer-reviewed journals were included. The following publication types were excluded: protocols, letter, editorial, commentary, unpublished manuscript, dissertation, government report, book and/or book chapter, conference proceeding, meeting abstract, lecture, and consensus development statements.

### Screening

All potentially relevant citations identified by the search strategy from the electronic databases were exported into EndNote X8 (Clarivate Analytics, Philadelphia, USA) for reference management and tracking of the screening process. A standardised Microsoft Excel (Microsoft Corporation, Redmond, USA) spreadsheet was used to enter results from the screening process. For the first level of screening, two reviewers (DT and MR) independently screened the titles and abstracts for all relevant and possibly relevant citations. In the second level of screening, the same reviewers independently reviewed full texts for all relevant and possibly relevant citations previously identified. Any disagreements during any phase of screening were resolved by discussion. If consensus could not be reached after discussion, a third reviewer (CC) was consulted to determine eligibility.

### Critical appraisal of the literature

Eligible articles were independently appraised for risk of bias by two reviewers (DT and MR) using the Scottish Intercollegiate Guidelines Network (SIGN) criteria for cohort studies [20, 21]. No relevant randomised controlled trials or case–control studies were identified. The SIGN criteria for cohort studies prompted us to qualitatively assess items that could contribute to selection, information, and confounding bias. The SIGN criteria were used to assist reviewers in making an informed overall judgement of the internal validity of studies. In accordance with the SIGN criteria, articles were rated as either high, acceptable, or unacceptable quality. If confounding was not considered, but other relevant items were done sufficiently well, the studies were rated as “acceptable” and the studies were accepted as association studies. Articles rated as high or acceptable quality were then deemed low risk of bias, while those rated as unacceptable quality were deemed high risk of bias. Discussion was used to solve disagreements and reach consensus among the two reviewers. A third reviewer (CC) was consulted if disagreements persisted.

### Data extraction, analysis, and synthesis

Data on study characteristics (e.g., author, year, study design, geographic region); participant characteristics (e.g., specific population, eligibility criteria), outcomes, risk factors assessed, and key findings from all eligible studies were extracted into a pre-piloted form by one reviewer (DT) and assessed for accuracy and completeness by another reviewer (CC). Data was extracted according to the CHARMS-PF (checklist for critical appraisal and data extraction for systematic reviews of prediction modelling studies—prognostic factors) where applicable [22]. We extracted measures of association between the risk factors and outcomes including odds ratios (OR), relative risks (RR), and hazard ratios (HR), and 95% confidence intervals (CI). If the confidence intervals were not reported, we computed them from the raw data if available. If a study included unadjusted and adjusted estimates, only adjusted estimates were extracted. Similar to previous reviews on risk factors for LBP [10, 11], risk factors were grouped into physical, sociodemographic, occupational, and psychological risk factors. They were also grouped by type of risk factor (e.g., marker, predictor, or determinant) [18].

A descriptive synthesis [23] was conducted due to the absence of adequate homogeneity across studies. We synthesised the associations between risk factors and LBP as (1) consistent association (association in the same direction demonstrated in ≥ 2 studies), (2) consistent non-association (no association demonstrated in ≥ 2 studies); (3) association/non-association (demonstrated in 1 study); and (4) inconsistent associations (≥ 2 studies demonstrating associations in different directions).

## Results

### Literature search

**Fig. 1:**
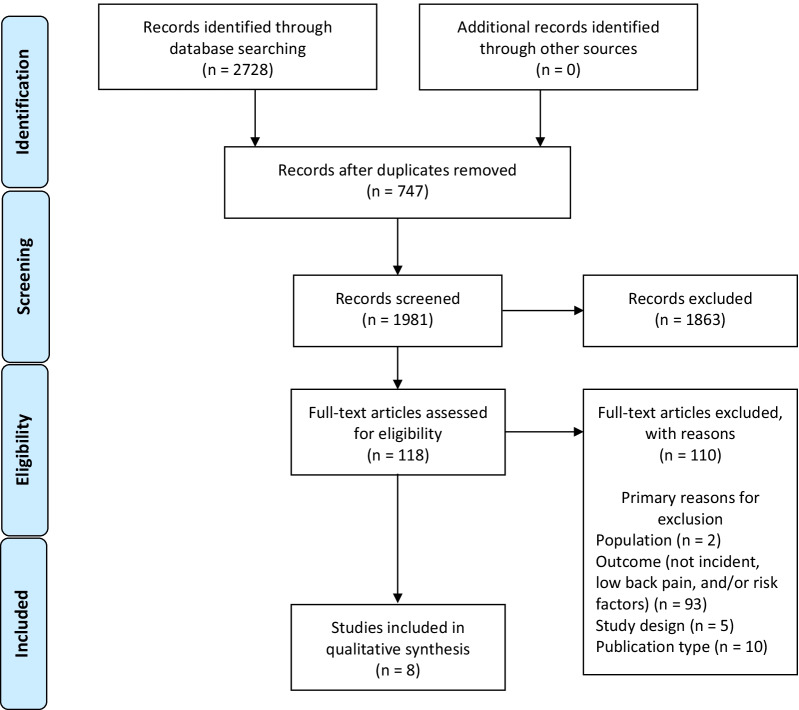
PRISMA flow diagram

We screened 1981 titles and abstracts for eligibility (Fig. 1). Of these, 118 full-texts were screened. We critically appraised eight eligible cohort studies [24–31] and all were deemed of acceptable quality (i.e., low risk of bias). No additional studies were found with hand-searching of reference lists of eligible studies.

### Risk of bias

The accepted studies had some methodological limitations (Table 1). For example, in most studies, it was unclear (and marked as “can’t say” according to the SIGN criteria) if the method of exposure assessment was reliable (6/8) [24–27, 30, 31] and there was no evidence that the method of outcome assessment was valid and/or reliable (6/8) [24, 25, 27, 29–31]. Additionally, it was unclear (“can’t say”) if the assessment of outcome was made blind to exposure status or if there was recognition that knowledge of exposure status could have influenced the assessment of outcome in all studies where these criteria were applicable (3/3) [27, 30, 31]. Potential confounders were not clearly identified in two studies [29, 31] that aimed to assess causal factors; therefore, we synthesised risk factors as risk markers rather than determinants.

**Table 1:** Risk of bias assessment

SIGN criteria	Roy and Lopez	Ernat et al.	MacGregor et al.	Knox et al.	Taanila et al.	Seay et al.	Zack et al.	Monnier et al.
1.1 Appropriate and clearly focused question	Y	Y	Y	Y	Y	Y	Y	Y
1.2 Groups are comparable in all respects	NA	Y	Y	Y	NA	Y	CS	NA
1.3 Reports participation rates of each group	Y	NA	NA	NA	Y	NA	NA	Y
1.4 Likelihood that subjects had outcome at time of enrolment taken into account in analysis	Y	Y	Y	Y	Y	Y	Y	Y
1.5 Reports dropout/withdrawal rates	Y, 4.6%	NA	NA	NA	Y, 31%	NA	NA	Y, 3.8%
1.6 Compares full participants with those lost to follow-up	N	NA	NA	NA	NA	NA	NA	Y
1.7 Outcomes are clearly defined	Y	Y	Y	Y	Y	Y	Y	Y
1.8 Assessment of outcome is made blind to exposure	NA	CS	NA	NA	NA	CS	CS	NA
1.9 Recognise that knowledge of exposure status could have influenced assessment of outcome	NA	CS	NA	NA	NA	CS	CS	NA
1.10 Method of assessment of exposure is reliable	Y	CS	CS	CS	CS	CS	CS	Y
1.11 Evidence that method of outcome assessment is valid and reliable	N	N	N	N	Y	N	CS	Y
1.12 Exposure/prognostic factor assessed more than once	NA	NA	NA	NA	NA	NA	NA	NA
1.13 Addresses main potential confounders	CS	Y	Y	Y	Y	Y	N	Y
1.14 Confidence intervals provided	Y	Y	Y	Y	Y	Y	Y	Y
2.1 Overall assessment of study based on risk of bias, clinical considerations, and evaluation of methodology	Acceptable	Acceptable	Acceptable	Acceptable	Acceptable	Acceptable	Acceptable	Acceptable

Y, Yes; NA, not applicable; N, No; CS, Can’t say

### Study characteristics

A summary of study characteristics is presented in Table 2. The majority of eligible studies were conducted in the U.S. (5/8) [24, 25, 27, 29, 30], and one each were conducted in Sweden [28], Finland [26], and Israel [31]. Two studies assessed Marines [24, 28], two assessed Army personnel [27, 29], and the remaining studies assessed the military as a whole [25, 26, 30, 31].

**Table 2:** Study characteristics

Author, year, country; study design/source; risk of bias	Population	Outcomes	Candidate risk factors; analysis; type of risk factor (marker/predictor/determinant); factors assessed
MacGregor et al., 2012, United States Historical cohort (pre-existing data) Low risk of bias	N = 36,680 (34,879 M, 95%) Sample size calculation: N/A Missing data: N/A Age: 18+ years Population: US Marines; identified from records from Defense Manpower Data Center Inclusion: First deployment to Operation Iraqi Freedom between January 2005 and November 2008; without second deployment within 365 days of the end-date of their first deployment Exclusion: Previous back-related diagnosis (ICD-9-CM 724 series); missing demographic information; died during deployment	New-onset diagnosis of LBP: presence of an ICD-9-CM code of 724.2 (lumbago) over the course of deployment or within 1 year of end of deployment F/u period: Over course of deployment (greater than 1 month and less than 18 months) or within 1 year of end of deployment	Age (18–24, ≥ 25 years); rank (junior, midlevel, senior); sex (male, female); location country of deployment (Iraq, Kuwait); time deployed (1–7 months, > 7 months); blast injury (no, yes); occupation (administrative/other, communications/intelligence, infantry, service/supply, electrical/mechanical/craftsworker) Multivariate logistic regression Risk marker Sociodemographic, occupational
Knox et al., 2014, United States Historical cohort (pre-existing data; comparison group: nonvehicle operators) Low risk of bias	N = 213,024 person-years (186,084 M, 87.4%) Sample size calculation: N/A Missing data: N/A Population: US Military; identified from Defense Medical Epidemiology Database Inclusion: Ambulatory first encounters during 1998–2006 for ICD-9 code 742.20 (LBP) Exclusion: Repeat coding of same initial diagnosis for multiple visits for LBP provided to a single service member during study period	First occurrence of ICD-9 code 724.20 (LBP) 1 exposure year defined as 1 year that the service member was exposed to the risk factors for LBP while enlisted F/u period: 8 years (database searched from 1998–2006)	Military vehicle operator; age (< 20, 20–29, 30–39, > 40); sex (male, female); race (black, white, other); marital status (single, married, other); rank group (junior, senior); military service (Army, Air Force, Marine) Multivariate Poisson regression Risk marker Sociodemographic, occupational
Taanila et al., 2012, Finland Prospective cohort Low risk of bias	N = 982 (982 M, 100%) Sample size calculation: Not reported Response rate: 1489/1513 (98%) Missing data: 304/982, 31% lost to follow-up Age (median): 19 years Population: Finnish Defence Forces; male conscripts; anti-tank, signal, mortar, engineer companies; data collected July 10, 2006–July 4, 2008) Inclusion: Young; healthy; male; medical checkup by a clinician during the 12 months before entering the military Exclusion: Female; ≥ 1 day of LBP during the last month before military entry; unanswered pre-information questionnaire; upper back pain	LBP occurring during active service hours, leisure time, or on the way to or from the garrison for leave ICD-10 diagnoses: M54 (dorsalgia), M54.5 (LBP), M41 (scoliosis), M54.9 (dorsalgia, unspecified), M54.3 (sciatica) Confirmed by physician based on computerised patient records F/u period: 6 months	Father’s occupation (not physical, physical, unemployed/retire); level of education (high, lower); degrees achieved in school (high, low or average); urbanisation level of place of residence (countryside, small population centre, midsize town or city, bigger city); age (18–20, 21–28); company (anti-tank, signal, mortar, engineer); body mass index (underweight, normal, pre-obese, obese); waist circumference thin, normal, increased, high); height (shortest quartile, second quartile, third quartile, tallest quartile); self-assessed health (good or very good, average or inferior); presence of chronic disease (no, yes); use of regular medication (no, yes); previous orthopedic surgery (never, yes); presence of chronic impairment or disability (no, yes); sports injury during the last month (no, yes); other MSK symptoms (minimal, mild, clear); smoking habits (never regularly, has smoked regularly); use of alcohol (< 1 time per month, 1–2 times per week, ≥ 3 times per week); frequency of drunkenness before military service (< 1 time per week, ≥ 1 time per week); agreeing that soldier needs good physical fitness (yes, no); frequency of sweating exercise per week (≥ 3 times per week, 1–2 times per week, only leisured exercise, no physical exercise); participation in individual aerobic sports (yes at least sometimes, no); belonging to a sports club (yes an active member, no); participation in competitive sports (yes, no); last degree in school sports (good or excellent, poor or fair); self-assessed physical fitness (good or very good, average or inferior); Cooper test (12-min running test) (excellent, good, fair, poor); pull-up test (excellent, good, fair, poor); standing long jump test (excellent, good, fair, poor); sit-up test (excellent, good, fair, poor); push-up test (excellent, good, fair, poor); back-lift test (excellent, good, fair, poor); combination of push-up and Cooper test (excellent, good, fair, poor); combination of back lift and Cooper test (excellent, good, fair, poor); combination of sit-up and push-up test (excellent, good, fair, poor); combination of push-up and back lift test (excellent, good, fair, poor) Multivariate Cox regression Risk marker Physical, sociodemographic, occupational
Seay et al., 2017, United States Historical cohort (pre-existing data; comparison group: no lower extremity injury) Low risk of bias	N = 1,066,535; average of 213,307 included in each of 5 yearly cohorts (959,721 M, 90%) Sample size calculation: N/A Missing data: N/A Population: US Army soldiers; identified from Total Army Injury and Health Outcomes Database Inclusion: Active duty from January 1, 2007 to December 31, 2011 Exclusion: History of lower-extremity MSK injury; history of LBP or back injury; nonmusculoskeletal back pain	Incident LBP identified by ICD-9-CM code (353.4, 720.2, 721.3, 721.42, 722.10, 722.52, 722.73, 722.93, 724.02, 724.03, 724.2, 724.3, 724.4, 724.5, 724.6, 738.4, 739.3, 739.4, 756.11, 756.12, 839.20, 846.1, 846.8, 846.9, 847.2, 847.3) F/u period: From January 1 of calendar year until either: (1) incident LBP; (2) end of active duty; (3) December 31 of respective year	Lower extremity injury (yes, no); sex (female, male) Extended Andersen–Gill Cox regression Risk marker Physical
Monnier et al., 2019, Sweden Prospective cohort Low risk of bias	N = 53 (48 M, 91%) Sample size calculation: Not reported Response rate: 53/56 (95%) Missing data: 2/53, 3.8% withdrew from course Age (mean (SD)): 21.8 years (3.4 years) Population: Marines in Swedish Armed Forces in training course Inclusion: Entering with intention to complete 4 month marine training course; not seeking medical care Exclusion: Ongoing LBP at baseline lasting ≥ 5 consecutive weeks adjacent to the course start	Occurrence of any self-rated pain in the lower back (from twelfth ribs to lower gluteal folds) within the preceding week, as reported during the weekly follow-up LBP limiting work ability: occurrence of any self-rated pain in the lower back within the preceding week that had limited work ability F/u period: Weekly, for 16 weeks	Body weight; body height (> 1.80 m, ≤ 1.80 m); back pain within 6 months prior to course start (no, yes); hip/knee pain within 6 months prior to course start (no, yes); neck/shoulder pain within 6 months prior to course start (no, yes); mental distress GHQ-12 score (< 4, ≥ 4); current work ability with regard to best ever (≥ 9, < 9); direct (within 3 months) from basic military training (no, yes); physical training sessions per week (> 2, ≤ 2); muscular strength training sessions per week (2–4, ≤ 1, ≥ 5); aerobic fitness training sessions per week (> 1, ≤ 1); kettlebell lift (kg x repetitions) (> 760, ≤ 760); number of pull-ups (≥ 4, ≤ 3); double leg lift and power (pass, fail); double leg lift and alternate leg extension (pass, fail) Andersen-Gill repeated time-to-event regression Risk marker Physical
Roy and Lopez, 2013, United States Prospective cohort Low risk of bias	N = 805 (approximately 758 M, 94%) Sample size calculation: Not reported Response rate: 1194/3500 (34%) Missing data: 55/1194, 4.6% medically evacuated Age: Brigade Support Battalion (27.7 ± 6.4 years); Brigade Special Troops Battalion (25.8 ± 4.9 years); Reconnaissance, Surveillance, and Target Acquisition Squadron (25.8 ± 5.6 years); Field Artillery (26.9 ± 6.2 years); Infantry Battalions (24.9 ± 4.9 years) Population: US Army Inclusion: All soldiers deploying as a member of the Brigade Combat Teams from June 2009–August 2010 Exclusion: Current LBP	Self-reported LBP: pain interfering with the performance of occupational tasks F/u period: 12 months	Number of hours per week spent on cardiovascular training, core training, and strength training; history of LBP (yes, no); hours per day or week spent on occupational tasks including wearing body armour, lifting objects weighing more than 30 lbs, dismounted patrolling, riding in tactical vehicles, or desk work; and average weight of equipment worn Logistic regression Risk marker Physical, occupational
Ernat et al., 2012, United States Historical cohort (pre-existing data; comparison group: noninfantry soldiers) Low risk of bias	N = 791,526 person-years of data Sample size calculation: N/A Missing data: N/A Population: US Military; infantrymen; identified from records from Defense Medical Epidemiology Database Inclusion: Junior (E1–E4) and senior (E5–E9) enlisted infantry members Exclusion: Officers	ICD-9-CM code 724.20 for ambulatory patients’ initial visits for “lumbago” (LBP) F/u period: 8 years (database searched from 1998–2006)	Infantrymen; age (< 20, 20–24, 25–29, 30–34, 35–39, > 40); marital status (single married, other); race (white, black, other); rank (junior, senior); and branch of military service (Army, Air Force, Marine) Multivariate Poisson regression Risk marker Sociodemographic, occupational
Zack et al., 2018, Israel Historical cohort (pre-existing data) Low risk of bias	N = 80,599 (80,599 M, 100%) total; 73,989 (73,989 M, 100%) with no history of LBP Sample size calculation: N/A Missing data: N/A Age (mean (SD)): 19.06 years (1.4 years) Population: Soldiers drafted to Israel Defense Forces Inclusion: Drafted between January 1, 1997 to December 31, 2006; served in administrative or driving professions for a full 36-months period during study period Exclusion: Not specified	Newly reported LBP evaluated by certified orthopedic surgeon in accordance with medical parameters defined in the military medical book of profiles (based on reported LBP, findings on physical examination, and radiologic findings) F/u period: 36 months	Occupational groups, consisting of administrative units, professional car drivers, and professional truck drivers Incidence and relative risk rates Risk marker Occupational

LBP, low back pain; N, number; M, male; F, female; response rate, number of participants enrolled in study/number of participants invited to study; US, United States; F/u, follow up; ICD-9-CM, International Classification of Diseases, 9th Revision, Clinical Modification; N/A, not applicable

All studies were cohort studies (5/8 single-group cohorts [24, 26, 28, 29, 31]), which assessed risk factors for incident LBP in the active military population. Three studies were prospective cohort studies [26, 28, 29], while five were historical cohort studies conducted using pre-existing administrative and/or clinical data [24, 25, 27, 30, 31]. All studies examined non-causal associations between candidate risk factors and incident LBP, as there were either no clear a priori variables defined as potentially important for predicting incident LBP by the studies or the necessary confounding variables were not identified a priori and 1controlled for; therefore, only risk markers were identified. No studies identified included prediction or causal modelling; therefore, risk predictors and risk determinants could not be identified. Half of the studies examined risk factors in more than one category (e.g., physical, sociodemographic, and/or occupational) [24–26, 29]. Four studies examined physical risk factors (e.g., physical fitness, body characteristics) [26–29], three studies examined sociodemographic risk factors (e.g., age, sex, education) [24–26], and six studies examined occupational risk factors (e.g., occupational tasks, military service) [24–26, 29–31]. No studies assessed psychological risk factors for LBP.

### Overview of risk factors

In the eight studies included in our review, 37 risk factors (all risk markers) were examined: 13 physical factors, 16 sociodemographic factors, and 8 occupational factors. Among prospective cohort studies, all used self-reported questionnaires to identify the risk factors [26, 28, 29]. The historical cohort studies used administrative data to identify the risk factors [24, 25, 27, 30, 31]. There were no consistent confounding variables that were adjusted for by all studies; however, age (5/8) [24–27, 30] and sex (4/8) [24, 25, 27, 28] were most commonly adjusted for. The outcomes and key findings for each risk factor studied is presented in Table 3.

**Table 3:** Key findings by risk factors

Risk factor	Study	Outcomes and key findings
Physical factors (consistent associations)		
History of LBP	Monnier et al. (2019)	Incident LBP: Back pain within 6 months prior to course start (ref: no): Yes^1^: HR 2.47 (1.41; 4.31)* Incident LBP limiting work ability: Back pain within 6 months prior to course start (ref: no): Yes^2^: HR 3.58 (1.44; 8.90)*
	Roy and Lopez (2013)	Brigade Support Battalion: History of LBP (ref: no): Yes: OR 5.03 (1.61; 15.72)* Brigade Special Troops Battalion: History of LBP (ref: no): Yes: OR 8.91 (1.71; 46.46)* Infantry Battalions: History of LBP (ref: no): Yes: OR 2.20 (1.2; 4.04)*
Previous injury	Taanila et al. (2012)	Sports injury during last month (ref: no): Yes^3^: HR 1.7 (1.0; 2.8)*
	Seay et al. (2017)	Lower extremity injury^4^ (ref: no lower extremity injury): HR 1.70 (1.66; 1.74)* Pooled TR 0.90 (0.90; 0.91)* Males with lower extremity injury^4^ (ref: males with no lower extremity injury): HR 1.76 (1.72; 1.80)* Pooled TR 0.90 (0.89; 0.90)* Females with lower extremity injury^4^ (ref: females with no lower extremity injury): HR 1.43 (1.36; 1.50)* Pooled TR 0.93 (0.92; 0.94)*
Time spent on physical training	Monnier et al. (2019)	Incident LBP limiting work ability: Physical training sessions per week (ref: > 2): ≤ 2^2^: HR 2.96 (1.19; 7.39)*
	Roy and Lopez (2013)	Brigade Special Troops Battalion: Strength training (ref: less): More: OR 0.88 (0.78; 0.99)*
Physical factors (non-associations, single study)		
Body mass index	Taanila et al. (2012)	BMI (ref: normal 18.5 ≤ BMI < 25.0): Underweight BMI < 18.5^3^: HR 0.2 (0.0; 1.3) Pre-obese 25.0 ≤ BMI ≤ 30.0^3^: HR 0.9 (0.6; 1.3) Obese BMI ≥ 30.0^3^: HR 1.4 (0.8; 2.4)
Waist circumference	Taanila et al. (2012)	WC (ref: normal 80 ≤ WC < 94): Thin WC < 80^3^: HR 0.8 (0.5; 1.4) Increased 94 ≤ WC < 102^3^: HR 1.3 (0.8; 2.0) High WC ≥ 102^3^: HR 1.3 (0.7; 2.4)
Self-assessed health	Taanila et al. (2012)	Self-assessed health (ref: good or very good): Average or inferior^3^: HR 1.1 (0.8; 1.6)
Chronic disease	Taanila et al. (2012)	Chronic disease (ref: no): Yes^3^: HR 1.2 (0.8; 1.7)
Regular medications	Taanila et al. (2012)	Regular medication (ref: no): Yes^3^: HR 1.4 (0.9; 2.3)
Orthopedic surgery	Taanila et al. (2012)	Orthopedic surgery (ref: never): Yes^3^: HR 1.6 (0.9; 2.6)
Chronic impairment due to prior MSK injury	Taanila et al. (2012)	Chronic impairment or disability because of prior MSK injury (ref: no): Yes^3^: HR 1.4 (0.9; 2.2)
Self-assessed physical fitness	Taanila et al. (2012)	Self-assessed physical fitness (ref: good or very good): Average or inferior^3^: HR 1.3 (0.8; 1.9)
Physical factors (inconsistent associations)		
Poor performance on physical fitness tests	Monnier et al. (2019)	Incident LBP: Pull-up (number of repetitions) (ref: ≥ 4): ≤ 3^5^: HR 1.87 (1.17; 3.01)*
	Taanila et al. (2012)	Pull-up test (consecutive repeats without time limit) (ref: excellent ≥ 14): Good ≥ 10^3^: HR 1.6 (0.8; 3.1) Fair ≥ 6^3^: HR 1.3 (0.7; 2.5) Poor < 6^3^: HR 1.2 (0.7; 2.3) Standing long jump test (two attempts, best result observed) (ref: excellent ≥ 240 m): Good ≥ 220 m^3^: HR 0.9 (0.5; 1.5) Fair ≥ 200 m^3^: HR 1.1 (0.7; 1.7) Poor < 200 m^3^: HR 0.9 (0.5; 1.5) Sit-up test (repeats/60 s) (ref: excellent ≥ 48): Good ≥ 40^3^: HR 1.5 (0.8; 2.8) Fair ≥ 32^3^: HR 1.4 (0.8; 2.4) Poor < 32^3^: HR 1.7 (0.9; 3.0) Push-up test (repeats/60 s) (ref: excellent ≥ 38): Good ≥ 30^3^: HR 1.3 (0.8; 2.1) Fair ≥ 22^3^: HR 1.2 (0.8; 1.9) Poor < 22^3^: HR 1.6 (1.0; 2.6) Back-lift test (repeats/60 s) (ref: excellent ≥ 60): Good ≥ 50^3^: HR 1.2 (0.8; 1.9) Fair ≥ 40^3^: HR 1.2 (0.7; 1.8) Poor < 40^3^: HR 1.6 (0.9; 2.8) Combination of push-up and Cooper test (ref: excellent): Good^3^: HR 1.4 (0.8; 2.3) Fair^3^: HR 1.5 (0.8; 2.5) Poor^3^: HR 2.1 (1.1; 4.2)* Combination of back lift and Cooper test (ref: excellent): Good^3^: HR 1.3 (0.8; 2.1) Fair^3^: HR 1.5 (0.9; 2.5) Poor^3^: HR 2.4 (1.1; 5.4)* Combination of sit-up and push-up test (ref: excellent): Good^3^: HR 1.5 (0.8; 2.8) Fair^3^: HR 1.6 (0.9; 3.0) Poor^3^: HR 2.2 (1.1; 4.5)* Combination of push-up and back lift test (ref: excellent): Good^3^: HR 1.4 (0.8; 2.2) Fair^3^: HR 1.3 (0.8; 2.0) Poor^3^: HR 2.8 (1.4; 5.9)*
Height	Monnier et al. (2019)	Incident LBP: Body height (ref: > 1.80 m): ≤ 1.80m^1^: HR 1.98 (1.19; 3.29)* Incident LBP limiting work ability: Body height (ref: > 1.80 m): ≤ 1.80m^2^: HR 4.48 (2.01; 9.97)*
	Taanila et al. (2012)	Height (cm) (ref: shortest quartile ≤ 176): Second quartile 177–180^3^: HR 1.2 (0.7; 1.8) Third quartile 181–185^3^: HR 0.9 (0.5; 1.4) Tallest quartile ≥ 185^3^: HR 1.1 (0.7; 1.8)
Sociodemographic factors (consistent associations)		
Female	MacGregor et al. (2012)	Sex (ref: male): Female^6^: OR 1.94 (1.61; 2.34)*
	Knox et al. (2014)	Sex^#^ (ref: male): Female^7^: IRR 1.45 (1.39; 1.52)*
Sociodemographic factors (associations, single study)		
‘Single’ marital status	Knox et al. (2014)	Marital status^#^ (ref: married): Single^8^: IRR 0.87 (0.84; 0.91)* Other^8^: IRR 1.01 (0.91; 1.12)
Lower education level	Taanila et al. (2012)	Level of education (ref: higher—secondary school graduates, polytechnic, university student): Lower—comprehensive or vocational school)^3^: HR 1.6 (1.1; 2.3)* Degrees achieved in school (ref: high): Low or average^3^: HR 1.5 (1.0; 2.2)*
Sociodemographic factors (non-associations)		
Father’s occupation	Taanila et al. (2012)	Father’s occupation (ref: not physical): Physical^3^: HR 1.2 (0.8; 1.9) Unemployed or retired^3^: HR 1.4 (0.9; 2.2)
Urbanisation level of place of residence	Taanila et al. (2012)	Urbanisation level of place of residence (ref: countryside): Small population centre^3^: HR 1.1 (0.6; 2.0) Midsize town or city^3^: HR 1.0 (0.6; 1.7) Bigger city^3^: HR 1.2 (CI 0.7; 2.0)
Smoking habits	Taanila et al. (2012)	Smoking habits (ref: never regularly): Smoked regularly^3^: HR 1.1 (0.8; 1.6)
Use of alcohol	Taanila et al. (2012)	Use of alcohol (ref: < 1 time/month): 1–2 times/week^3^: HR 0.8 (0.5; 1.2) ≥ 3 times/week^3^: HR 0.6 (0.3; 1.2)
Frequency of drunkenness before military service	Taanila et al. (2012)	Frequency of drunkenness before military service (ref: < 1 time/week): ≥ 1 time/week^3^: HR 0.7 (0.5; 1.1)
Agreeing that soldiers need good physical fitness	Taanila et al. (2012)	Agrees that soldier needs good physical fitness (ref: yes): No^3^: HR 1.0 ( 0.7; 1.4)
Amount of time spent on sweating exercises	Taanila et al. (2012)	Sweating exercise (brisk leisure time sport) (ref: ≥ 3 times/week): 1–2 times/week^3^: HR 0.7 (0.5; 1.1) Only leisured exercise^3^: HR 1.3 (0.8; 2.1) No physical exercise^3^: HR 1.0 (0.6; 1.7)
Participation in individual aerobic sports	Taanila et al. (2012)	Participates in individual aerobic sports (ref: yes, at least sometimes): No^3^: HR 1.1 ( 0.7; 1.5)
Belonging to a sports club	Taanila et al. (2012)	Belongs to a sports club (ref: yes, active member): No^3^: HR 1.1 ( 0.7; 1.8)
Participation in competitive sports	Taanila et al. (2012)	Participates in competitive sports (ref: yes): No^3^: HR 1.1 ( 0.7; 1.7)
Last degree in school sports	Taanila et al. (2012)	Last degree in school sports (ref: good or excellent): Poor or fair^3^: HR 0.8 (0.5; 1.3)
Race	Knox et al. (2014)	Race^#^ (ref: other): Black^9^: IRR 1.07 (1.00; 1.14) White^9^: IRR 1.06 (1.00; 1.13)
Sociodemographic factors (inconsistent associations)		
Age	Knox et al. (2014)	Age^#^ (ref: 30–39): < 20^10^: IRR 1.24 (1.15; 1.36)* 20–29^10^: IRR 0.96 (0.91; 1.01) > 40^10^: IRR 1.23 (1.0; 1.38)
	Ernat et al. (2012)	Infantrymen stratified by age (ref: control): < 20^11^: IRR 0.61 (0.59; 0.63)* 20–29^11^: IRR 0.66 (0.65; 0.67)* 30–39^11^: IRR 0.86 (0.83; 0.88)* > 40^11^: IRR 0.91 (0.86; 0.97)*
	Taanila et al. (2012)	Age (ref: 18–20): 21–28^3^: HR 1.8 (1.0; 3.4)
	MacGregor et al. (2012)	Age (ref: 18–24): ≥ 25^6^: OR 1.13 (0.94, 1.36)
Occupational factors (consistent associations)		
Lower rank	Knox et al. (2014)	Rank^#^ (ref: senior E5–E9): Junior E1–E4^12^: IRR 1.60 (1.52; 1.70)*
	MacGregor et al. (2012)	Rank (ref: junior E1–E3): Midlevel E4–E5^6^: OR 0.73 (0.64; 0.83)* Senior E6–E9^6^: OR 0.98 (0.76; 1.26)
Occupational factors (associations, single study)		
Blast injury	MacGregor et al. (2012)	Blast injury (ref: no): Yes^6^: OR 2.29 (1.64; 3.19)*
Job duties	Roy and Lopez (2013)	Brigade Support Battalion: Lifting objects (ref: < 30 lbs): > 30 lbs: OR 1.30 (1.06; 1.60)* Brigade Special Troops Battalion: Body armour (ref: no): Yes: OR 1.23 (1.03; 1.47)* Reconnaissance, Surveillance, and Target Acquisition Squadron: Body armour (ref: no): Yes: OR 1.30 (1.11; 1.53)* Infantry Battalions: Body armour (ref: no): Yes: OR 1.14 (1.07; 1.21)*
Service type	Knox et al. (2014)	Military service^#^ (ref: Marine): Army^13^: IRR 2.74 (2.60; 2.89)* Air Force^13^: IRR 1.98 (1.84; 2.14)*
Occupational factors (non-associations, single study)		
Location of deployment	MacGregor et al. (2012)	Location country (ref: Iraq): Kuwait^6^: OR 1.11 (1.00; 1.24)
Time deployed	MacGregor et al. (2012)	Time deployed (ref: 1–7 months): > 7 months^6^: OR 1.06 (0.95; 1.19)
Occupational factors (inconsistent associations)		
Military occupations	MacGregor et al. (2012)	Occupation (ref: administrative/other): Communications/intelligence^1^: OR 0.82 (0.64; 1.06) Infantry^6^: OR 0.86 (0.73; 1.02) Service/supply^6^: OR 1.33 (1.12; 1.59)* Electrical/mechanical/craftsworker^1^: OR 1.31 (1.12; 1.53)*
	Taanila et al. (2012)	Company (ref: anti-tank): Signal^3^: HR 1.4 (0.9; 2.3) Mortar^3^: HR 1.0 ( 0.5; 1.8) Engineer^3^: HR 2.0 (CI 1.2; 3.3)*
	Ernat et al. (2012)	Infantrymen^11^ (ref: control): IRR 0.69 (95% CI 0.68; 0.70)* Infantrymen stratified by age (ref: control): < 20^11^: IRR 0.61 (0.59; 0.63)* 20–29^11^: IRR 0.66 (0.65; 0.67)* 30–39^11^: IRR 0.86 (0.83; 0.88)* > 40^11^: IRR 0.91 (0.86; 0.97)* Infantrymen stratified by rank (ref: control): Junior: Unadjusted IRR 0.59 (no CI provided) Senior: Unadjusted IRR 0.80 (no CI provided) Infantrymen stratified by branches of service (ref: control): Army: Unadjusted IRR 0.70 (no CI provided) Marine: Unadjusted IRR 0.59 (no CI provided)
Driving	Knox et al. (2014)	Military vehicle operator^14^ (ref: other occupations): IRR 1.15 (1.13; 1.17)*
	Zack et al. (2018)	Occupational categories (ref: administrative): Car drivers: RR 1.0 (0.79; 1.28) Truck drivers: RR 0.49 (0.40; 0.60)*

ref, referent category; CI, confidence interval; OR, odds ratio; IRR, incidence rate ratio; ICD-10, International Classification of Diseases, 10th Revision; HR, hazard ratio; BMI, body mass index (kg/m^2^); WC, waist circumference (cm); MSK, musculoskeletal; m, metres; s, seconds; TR, time ratio; GHQ, General Health Questionnaire; lbs, pounds; RR, relative risk

Variables adjusted for: ^1^Sex; ^2^Sex, neck/shoulder pain previous to course start; ^3^Age, company, smoking, baseline medical conditions (sports injury, sum factor of earlier musculoskeletal symptoms, regular medication, chronic impairment or disability because of prior musculoskeletal injury, orthopedic surgery), education level, school degree level, father’s occupation, participating in individual aerobic sports; ^4^Sex, age, race, rank, time in service, military occupation specialty physical demands; ^5^Prior back pain, body height; ^6^Age, rank, sex, location country, time deployed, blast injury, occupation; ^7^Age, race, rank, service, marital status; ^8^Age, sex, race, rank, service; ^9^Age, sex, rank, marital status, service; ^10^Sex, service, rank, marital status, race; ^11^Age, race, rank, service, marital status; ^12^Age, sex, race, marital status, service; ^13^Age, sex, race, marital status, rank; ^14^Sex, race, rank, service, marital status

*Statistically significant; ^#^among vehicle operators in the US military

### Consistent associations between physical risk factors and LBP

A history of LBP demonstrated a consistent association with LBP during active duty military service [28, 29]. Monnier et al. concluded that back pain within six months prior was a risk factor for both LBP (HR 2.47, 95% CI 1.41–4.31) and LBP limiting work ability (HR 3.58, 95% CI 1.44–8.90) [28] and Roy and Lopez concluded that a history of LBP prior to military service was associated with LBP in the Brigade Support Battalion (OR 5.03, 95% CI 1.61–15.72), the Brigade Special Troops Battalion (OR 8.91, 95% CI 1.71–46.46), and the Infantry Battalion (OR 2.20, 95% CI 1.2–4.04) compared to those without a history of LBP [29]. Similarly, previous injury (e.g., lower extremity injury or sports injury) consistently demonstrated an association with LBP [26, 27]. Taanila et al. [26] concluded that having a sports injury during the prior month (HR 1.7, 95% CI 1.0–2.8) was a risk factor for LBP, while Seay et al. [27] concluded that lower extremity injury was a risk factor for LBP (HR 1.70, 95% CI 1.66–1.74) irrespective of sex (males—HR 1.76, 95% CI 1.72–1.80; females—HR 1.43, 95% CI 1.36–1.50). The amount of time spent on physical training also had an association with LBP, with one study demonstrating that those participating in fewer physical training sessions per week had a greater risk of LBP limiting work ability than those participating in more physical training sessions per week (HR 2.96, 95% CI 1.19–7.39) [28], while another study demonstrated that participation in more strength training was associated with a lower risk for LBP (OR 0.88, 95% CI 0.78–0.99) [29].

### Non-associations between physical risk factors and LBP

Based on a single study by Taanila et al., the following factors were not found to be associated with LBP: body mass index, waist circumference, self-assessed health, chronic disease, regular medications, orthopedic surgery, chronic impairment due to prior MSK injury, and self-assessed physical fitness [26].

### Inconsistent associations between physical risk factors and LBP

There was conflicting evidence on whether poor performance on various physical fitness tests were associated with LBP. For example, Monnier et al. demonstrated that performing less pull ups was associated with incident LBP (HR 1.87, 95% CI 1.17–3.01) [28], but Taanila et al. found no association [26]. Similarly, Taanila et al. found an association between poor performance on certain combinations of physical fitness tests (e.g., poor results in the combination of push-up and Cooper test (12-min running test) (HR 2.1, 95% CI 1.1–4.2); poor results in the combination of back lift and Cooper test (HR 2.4, 95% CI 1.1–5.4); poor results in the combination of sit-up and push-up test (HR 2.2, 95% CI 1.1–4.5); and poor results in the combination of push-up and back lift test (HR 2.8, 95% CI 1.4–5.9)) but not individual physical fitness tests (e.g., push-up or Cooper test alone) [26]. There was also conflicting evidence on the association between height and LBP, with one study reporting an association between shorter height and LBP (HR 1.98, 95% CI 1.19–3.29) and LBP limiting work ability (HR 4.48, 95% CI 2.01–9.97) [28], while another study found no association [26].

### Consistent associations between sociodemographic risk factors and LBP

Being female was the only sociodemographic risk factor that consistently demonstrated an association with LBP [24, 25]. MacGregor et al. concluded that females (OR 1.94, 95% CI 1.61–2.34) were more likely to report LBP compared to males [24], and Knox et al. concluded that being female (IRR 1.45, 95% CI 1.39–1.52) was associated with LBP [25].

### Associations between sociodemographic risk factors and LBP

An association with LBP was reported for ‘single’ marital status being less likely to experience LBP (IRR 0.87, 95% CI 0.84–0.91) compared to individuals reporting ‘married’ as their marital status [25]. Additionally, lower education level (HR 1.6, 95% CI 1.1–2.3) was associated with LBP [26]. As these were only reported in single studies, these sociodemographic risk factors may be further studied.

### Non-associations between sociodemographic risk factors and LBP

Based on a single study by Taanila et al., the following factors were not found to be associated with LBP: father’s occupation, urbanisation level of place of residence, smoking habits, use of alcohol, frequency of drunkenness before military service, agreeing that soldiers need good physical fitness, amount of time spent on sweating exercises, participation in individual aerobic sports, belonging to a sports club, participation in competitive sports, and last degree in school sports [26]. Additionally, Knox et al. reported that race was not associated with LBP [25].

### Inconsistent associations between sociodemographic risk factors and LBP

There was conflicting evidence on the association between age and LBP, with Knox et al. reporting that younger age (less than 20 years) was associated with LBP (IRR 1.24, 95% CI 1.15–1.36) [25], while Ernat et al. reported that among infantrymen, the incidence of LBP increased with age (from IRR 0.61, 95% CI 0.59–0.63 in those under the age of 20 to IRR 0.91, 95% CI 0.86–0.97 in those over the age of 40) [30]. Two studies reported no association between age and LBP [24, 26].

### Consistent associations between occupational risk factors and LBP

Among occupational risk factors, lower rank consistently demonstrated an association with LBP, with one study demonstrating that junior rank was associated with a higher risk for incident LBP compared to those with senior rank (IRR 1.60, 95% CI 1.52–1.70) [25], while another study demonstrated that mid-level ranks (compared to junior ranks) were associated with a lower risk for LBP (OR 0.73, 95% CI 0.64–0.83) [24].

### Associations between occupational risk factors and LBP

Several risk factors demonstrating an association with LBP were studied in single studies. These included having a blast injury (OR 2.29, 95% CI 1.64–3.19) [24], job duties (e.g., lifting > 30 pounds (OR 1.30, 95% CI 1.06–1.60) or wearing body armour (OR 1.14–1.30, 95% CI 1.07–1.53)) [29], and service type (e.g., Army (IRR 2.74, 95% CI 2.60–2.89) and Air Force (IRR 1.98, 95% CI 1.84–2.14) compared to Marines) [25]. In a study of U.S. military service members, no association with LBP was found for location of deployment and time deployed [24].

### Inconsistent associations between occupational risk factors and LBP

There were no military occupations that were consistently found to be associated with developing LBP, as positive associations were found for many different occupations [24, 26, 30]. MacGregor et al. concluded that being in the service/supply occupation (compared to administrative/other occupations) (OR 1.33, 95% CI 1.12–1.59) and being in the electrical/mechanical/craftsworker occupation (compared to administrative/other occupations) (OR 1.31, 95% CI 1.12–1.53) were risk factors for LBP [24]. Taanila et al. concluded that being part of the engineer company (HR 2.0, 95% CI 1.2–3.3) was associated with LBP compared to those working in the anti-tank company [26]. Ernat et al. concluded that infantrymen had a lower risk of LBP compared to non-infantry soldiers (IRR 0.69, 95% CI 0.68–0.70) [30]. There was also no consistent evidence for the association of driving and incident LBP [25, 31]. Knox et al. concluded that being a military vehicle operator was associated with an increased risk of LBP compared to those of other occupations (IRR 1.15, 95% CI 1.13–1.17) [25], while Zack et al. concluded that professional truck drivers were less likely to experience LBP compared to those working in administrative units (RR 0.49, 95% CI 0.40–0.60) [31].

## Discussion

The objective of our systematic review was to synthesise the literature on risk factors of incident LBP in active duty military personnel. We identified eight relevant cohort studies. None of the studies were designed to assess a causal relationship between candidate factors and incident LBP; therefore, all studies identified risk markers. In active duty military personnel, we found consistent associations between LBP and physical factors (e.g., prior LBP, prior musculoskeletal injury, less time spent on physical training), sociodemographic factors (e.g., female sex), and occupational factors (e.g., lower rank). The magnitude of the associations between prior LBP and incident LBP ranged from 2.20 (95% CI 1.2–4.04) to 8.91 (95% CI 1.71–46.46) [28, 29]. We also found associations between LBP and other sociodemographic and occupational factors (e.g., married marital status, lower education level, blast injuries, job duties including lifting > 30 pounds or wearing body armour, Army or Air Force service type); non-associations between LBP and physical (e.g., body mass index, waist circumference, self-assessed health) and sociodemographic factors (e.g., race, smoking habits, urbanisation of place of residence); and inconsistent associations between LBP and other physical (e.g., poor performance on physical fitness tests), sociodemographic (e.g., younger age), and occupational (e.g., occupation types such as service/supply, electrical/mechanical/craftsworker, engineer, infantry, or military vehicle operators) factors. Psychological risk factors were not assessed in any of the included studies.

To our knowledge, no other reviews investigated risk factors for incident LBP in the military population; thus, it is unknown if our results are comparable. However, the risk factors identified in our review are comparable to risk factors identified in the literature for incident LBP in other occupational settings. Having previous episodes of LBP has been consistently shown to significantly increase the risk of new episodes of LBP in both community and occupational settings [2, 10, 11]. A systematic review and meta-analysis on incidence and risk factors for first-time LBP by Taylor et al. [10] indicated that physical risk factors for incident LBP (pain free at baseline) included increased weight or body mass index, poor health behaviours, a low assessment of physical fitness (e.g. measured on endurance or strength tests), and having occupational demands that include lifting or carrying more than 25 pounds. In an umbrella review of systematic reviews on risk factors for LBP [11], lifting over 25 kg, higher frequency of lifting, and prolonged standing or walking were also identified as risk factors for LBP.

In contrast to findings from other LBP reviews, no studies included in our review examined psychological or psychosocial risk factors for incident LBP among active duty military personnel. Psychosocial factors have also been found to increase the risk of developing LBP [2, 10, 11]. These include mental distress (e.g., feeling stressed, nervous, tense), depression, psychosomatic factors, sleep problems, job dissatisfaction and dissatisfaction with life, participation in monotonous work, and interpersonal stress at work [10, 11]. Many of these psychosocial risk factors were identified within occupational settings including workers in clerical support and office, agricultural and forestry, crafts and trades, machine operators, farming, manufacturing, and healthcare; however, these were not identified in our review. A potential reason for the lack of studies examining psychosocial risk factors for incident LBP among this population relates to the stigma-related barriers to help-seeking for mental health problems among military members (e.g., shame/embarrassment, negative social judgement, confidentiality concerns, employment-related discrimination) [32].

### Strengths and limitations

There is the possibility of publication bias in our review because we only included articles that were published in peer-reviewed journals and in English. Therefore, other potentially eligible articles may have been missed and non-English studies may be captured in a subsequent review. A major strength of our review was the comprehensive search strategy. We included six databases using a robust and peer-reviewed search strategy.

### Implications

Our study identifying only risk markers of incident LBP has research implications. For example, the markers that had a consistent association with LBP can be studied further to assess if they are predictors or determinants of LBP. Subsequently, strategies or interventions targeting identified modifiable risk predictors or determinants may then be developed and tested to see if they prevent LBP in the active duty military. The association between psychological or psychosocial factors and incident LBP in the active duty military should also be further studied, as they have been identified to be significant risk factors for LBP in both the general population and in various occupational settings [10, 11]. Given that we only identified risk markers and studies of “acceptable” methodological quality, future research of high methodological quality may change our conclusions.

## Conclusion

Physical and occupational risk factors for incident LBP in active duty military were most commonly studied, with less focus on sociodemographic factors and none on psychological or psychosocial factors. A prior history of LBP, less physical training, previous injury, female sex, and lower rank consistently demonstrated an association with LBP. There was conflicting evidence of association for performance on physical fitness tests, body height, age, and various occupations, including driving. Our conclusions may change in light of future studies of higher methodological quality; future studies should explore the role of psychological/psychosocial risk factors in the development of LBP among active duty military personnel, and whether identified risk markers predict or cause incident LBP. Our results are relevant for researchers, active duty military personnel, and other decision makers who may be involved in developing strategies to reduce the risk of LBP in the active duty military population.

## Supplementary Information

**Additional file 1.** PRISMA 2009 Checklist.

**Additional file 2.** Sample search strategy in MEDLINE (EBSCO).

## Data Availability

The datasets used and/or analysed during the current study are available from the corresponding author on reasonable request.

## Abbreviations

MSK: Musculoskeletal
LBP: Low back pain
U.S.: United States
OR: Odds ratio
RR: Relative risk
HR: Hazard ratio
CI: Confidence interval
IRR: Incidence rate ratio
