# Supplementary material for: Risk factors for low back pain in active military personnel: a systematic review
Source: Chiropr Man Therap. 2021 Dec 30;29:52. doi: 10.1186/s12998-021-00409-x (PMC8719410; doi:10.1186/s12998-021-00409-x)
Supplement: Supplementary file 2 — Additional file 2. Sample search strategy in MEDLINE (EBSCO). [file 12998_2021_409_MOESM2_ESM.docx]

Appendix 2: Search strategy MEDLINE (EBSCO)

1. Epidemiology

1. MH Epidemiology
2. MH Epidemiologic Studies+
3. MH Epidemiologic Factors+
4. MH Incidence
5. MH Causality+
6. MH Disability Evaluation
7. MH Risk Factors
8. MH Time Factors
9. MH Age Factors
10. MH Socioeconomic Factors
11. MH Sex Factors
12. MH Occupations
13. MH Educational Status
14. MH Comorbidity
15. MH Confounding Factors (Epidemiology)
16. MH Task Performance and Analysis
17. MH Physical Fitness+
18. MH Lifting
19. MH "Wounds and Injuries+
20. MH Stress, Psychological
21. MH Stress Disorders, Traumatic, Acute
22. MH Stress Disorders, Post-Traumatic
23. MH Recurrence
24. MH Psychology
25. MH Mental Health
26. MH Depression
27. MH Anxiety
28. MH Anxiety Disorders +
29. MH Mental Disorders +
30. MH Fear
31. MH Biochemistry
32. MH Biomarkers
33. MH Brain Chemistry
34. TI risk* or AB risk*
35. TI expos* or Ab expos*
36. TI epidemiol* or AB epidemiol*
37. TI (comorbid* or co-morbid*) or AB (comorbid* or co-morbid*)
38. TI (cope or copes or coping*) or AB (cope or copes or coping*)
39. TI (driving* or driver*) or AB (driving* or driver*)
40. TI (mental* n2 health*) or AB (mental* n2 health*)
41. TI (pre-exist* or preexist*) or AB (pre-exist* or preexist*)
42. TI (psychosocial* or psycho-social* or biopsychosocial*) or AB (psychosocial* or psycho-social* or biopsychosocial*)
43. TI (rate or rates) or AB (rate or rates)
44. TI (sociodemograph* or socio-demograph*) or AB (sociodemograph* or socio-demograph*)
45. TI (socioeconom* or socio-econom*) or AB (socioeconom* or socio-econom*)
46. TI age n2 factor* or AB age n2 factor*
47. TI anthropometr* or AB anthropometr*
48. TI anxiety* or AB anxiety*
49. TI associated* n2 factor* or AB associated* n2 factor*
50. TI biochem* or AB biochem*
51. TI biomarker* or AB biomarker*
52. TI body n2 size* or AB body n2 size*
53. TI causality* or AB causality*
54. TI cervical* n2 neuromuscular* or AB cervical* n2 neuromuscular*
55. TI co-morbid* or AB co-morbid*
56. TI comorbid* or AB comorbid*
57. TI confound* n2 factor* or AB confound* n2 factor*
58. TI confound* n2 factor* or AB confound* n2 factor*
59. TI coping* or AB coping*
60. TI danger* or AB danger*
61. TI data or AB data
62. TI demograph* or AB demograph*
63. TI depress* or AB depress*
64. TI determinant* or AB determinant*
65. TI disability* or AB disabilit*
66. TI duration* or AB duration*
67. TI education* or AB education*
68. TI environment* or AB environment*
69. TI etiolog* or AB etiolog*
70. TI expectation* or AB expectation*
71. TI factor* or AB factor*
72. TI fatigu* or AB fatigu*
73. TI fear* or AB fear*
74. TI fitness* or AB fitness*
75. TI g-force* or AB g-force* or TI gravitational* n2 force* or AB gravitational* n2 force*
76. TI gender* or AB gender*
77. TI genetic* or AB genetic*
78. TI goggle* or AB goggle*
79. TI heavy* n2 demand* or AB heavy* n2 demand*
80. TI heavy* n2 equipment* or AB heavy* n2 equipment*
81. TI helmet* or AB helmet*
82. TI history* or AB history*
83. TI incidence* or AB incidence*
84. TI informatic* or AB informatic*
85. TI injur* or AB injur*
86. TI job* or AB job*
87. TI lift* or AB lift*
88. TI load* or AB load*
89. TI neck* n2 guard* or AB neck* n2 guard*
90. TI neck* n2 strength* or AB neck* n2 strength*
91. TI neck* n2 stiff* or AB neck* n2 stiff*
92. TI number* n2 injur* or AB number* n2 injur*
93. TI occupation* or AB occupation*
94. TI pattern* or AB pattern*
95. TI physical* n2 demand* or AB physical* n2 demand*
96. TI population* or AB population*
97. TI predict* n2 factor* or AB predict* n2 factor*
98. TI predispos* or AB predispos* or TI pre-dispos* or AB pre-dispos*
99. TI prevent* or AB prevent*
100. TI previous* n2 episod* or AB previous* n2 episod*
101. TI protein* n2 enzym* or AB protein* n2 enzym*
102. TI psycholog* or AB psycholog*
103. TI PTSD* or AB PTSD*
104. TI range* n2 motion* or AB range* n2 motion*
105. TI rank* or AB rank*
106. TI (recur or recurs or recurrence*) or AB (recur or recurs or recurrence*)
107. TI sex or AB sex
108. TI social* or AB social*
109. TI societal* or AB societal*
110. TI statistic* or AB statistic*
111. TI stress* or AB stress*
112. TI task* or AB task* or TI (duty* or duties*) or AB (duty* or duties*)
113. TI time* or AB time*
114. TI trauma* or AB trauma*
115. TI trend* or AB trend*
116. MH Randomized Controlled Trial
117. MH Randomized Controlled Trials as Topic
118. MH Controlled Clinical Trials as Topic
119. MH Clinical Trials as Topic
120. MH Double-Blind Method
121. MH Single-Blind Method
122. MH Placebos
123. MH Cohort Studies
124. MH Follow-Up Studies
125. MH Longitudinal Studies
126. MH Prospective Studies
127. MH Retrospective Studies
128. MH Cross-Sectional Studies
129. MH Case-Control Studies+
130. PT Randomized Controlled Trial
131. PT Comparative Study
132. PT Controlled Clinical Trial
133. PT Clinical Trial
134. TI clinical n2 trial* or AB clinical n2 trial*
135. TI (random* or control* n2 trial* or comparative* n2 stud* or clinical* n1 trial*) or AB (random* or control* n2 trial* or comparative* n2 stud* or clinical* n1 trial*)
136. TI placebo* or AB placebo*
137. TI method* n2 (double* or single*) or AB method* n2 (double* or single*)
138. TI observational n2 (study or studies or analys*) or AB observational* n2 (study or studies or analys*)
139. TI cohort* or AB cohort*
140. TI case-control* or case* n2 control* or AB case-control* or case* n2 control*
141. TI longitudinal* or AB longitudinal*
142. TI prospective* or AB prospective*
143. TI retrospective* or AB retrospective*
144. TI retrospective* or AB retrospective*
145. TI cross-section* or AB cross-section* or TI cross n2 section* or AB cross n2 section*
146. TI (follow n2 up*) or follow-up* or followup* or followed-up*) or AB (follow n2 up*) or follow-up* or followup* or followed-up*)
147. TI comparative n2 (study or studies or analys*) or AB comparative n2 (study or studies or analys*)
148. TI survey* or AB survey*
149. OR /1-148

2. Low back Pain

1. MH Low Back Pain
2. MH Back Pain +
3. MH Back Injuries
4. MH Intervertebral Disc Degeneration
5. MH Intervertebral Disc Displacement
6. MH Osteoarthritis, Spine
7. MH Piriformis Muscle Syndrome
8. MH Polyradiculopathy
9. MH Sciatica
10. MH Spine
11. MH Spinal Diseases
12. MH Spinal Stenosis
13. MH Spinal Injuries
14. MH Spondylolysis
15. MH Synovial Cyst
16. MH Spinal Curvatures
17. MH Lumbar Vertebrae/ IN
18. MH Lumbosacral Region/ IN
19. MH Back Muscles+/IN
20. MH Coccyx/ IN
21. MH Zygapophyseal Joint/IN
22. MH Intervertebral Disc/IN
23. MH Lumbar Vertebrae/ injuries
24. MH Lumbosacral region/ injuries
25. MH Back Muscles/injuries
26. MH Coccyx/injuries
27. TI (low back n2 pain* or low-back pain* or low n2 back-pain*) or AB (low back n2 pain* or low-back pain* or low n2 back-pain*)
28. TI (low back n2 injur* or low-back injur* or low n2 back-injur*) or AB (low back n2 injur* or low-back injur* or low n2 back-injur*)
29. TI (low back n2 trauma* or low-back trauma* or low n2 back-trauma*) or AB (low back n2 trauma* or low-back trauma* or low n2 back-trauma*)
30. TI (lower back n2 pain* or lower-back pain* or lower n2 back-pain*) or AB (lower back n2 pain* or lower-back pain* or lower n2 back-pain*)
31. TI (lower back n2 injur* or lower-back injur* or lower n2 back-injur*) or AB (lower back n2 injur* or lower-back injur* or lower n2 back-injur*)
32. TI (lower back n2 trauma* or lower-back trauma* or lower n2 back-trauma*) or AB (lower back n2 trauma* or lower-back trauma* or lower n2 back-trauma*)
33. TI (lower trunk n2 injur* or lower-trunk injur* or lower n2 trunk-injur*) or AB (lower trunk n2 injur* or lower-trunk injur* or lower n2 trunk-injur*)
34. TI (trunk* n2 pain*) or AB (trunk* n2 pain*)
35. TI "lumbar disc" n2 (extru* or degenerat* or displac* or herniat* or prolaps* or sequest* or slipped* or protru* or avuls*) or AB "lumbar disc" n2 (extru* or degenerat* or displac* or herniat* or prolaps* or sequest* or slipped* or protru* or avuls*)
36. TI "lumbar disk" n2 (extru* or degenerat* or displac* or herniat* or prolaps* or sequest* or slipped* or protru* or avuls*) or AB "lumbar disk" n2 (extru* or degenerat* or displac* or herniat* or prolaps* or sequest* or slipped* or protru* or avuls*)
37. TI lumbar* n2 (pain* or facet* or nerve* n2 root* or osteoarthr* or radicul* or stenos* or spondylo* or zygapohys* or injur* or trauma* or discomfort* or dysfunction* or sore* or herniat*) or AB lumbar* n2 (pain* or facet* or nerve* n2 root* or osteoarthr* or radicul* or stenos* or spondylo* or zygapohys* or injur* or trauma* or discomfort* or dysfunction* or sore* or herniat*)
38. TI (back n2 ache*) or (back n2 injur*) or (back n1 trauma*) or (back n2 sprain*) or (back n2 strain*) or AB (back n2 ache*) or (back n2 injur*) or (back n1 trauma*) or (back n2 sprain*) or (back n2 strain*)
39. TI (backache* or back-ache*) or AB (backache* or back-ache*)
40. TI (back n2 pain* or back-pain*) or AB (back n2 pain* or back-pain*)
41. TI "intervertebral disc" n2 (pain* or extru* or degenerat* or displac* or herniat* or prolaps* or sequest* or slipped* or protru* or avuls*) or AB "intervertebral disc" n2 (pain* or extru* or degenerat* or displac* or herniat* or prolaps* or sequest* or slipped* or protru* or avuls*)
42. TI "intervertebral disk" n2 (pain* or extru* or degenerat* or displac* or herniat* or prolaps* or sequest* or slipped* or protru* or avuls*) or AB "intervertebral disk" n2 (pain* or extru* or degenerat* or displac* or herniat* or prolaps* or sequest* or slipped* or protru* or avuls*)
43. TI coccydynia or AB coccydynia
44. TI coccy* n2 pain* or AB coccy* n2 pain*
45. TI coccygalg* or AB coccygalg*
46. TI dorsalg* or AB dorsalg*
47. TI lumbo* n2 (pain* or facet* or nerve* n2 root* or osteoarthr* or radicul* or stenos* or spondylo* or zygapohys* or injur* or trauma* or discomfort* or dysfunction* or sore* or herniat*) or AB lumbo* n2 (pain* or facet* or nerve* n2 root* or osteoarthr* or radicul* or stenos* or spondylo* or zygapohys* or injur* or trauma* or discomfort* or dysfunction* or sore* or herniat*)
48. TI lumboischialgi* or AB lumboischialgi*
49. TI lumbago* or AB lumbago*
50. TI (piriformis* n2 syndrome*) or AB (piriformis* n2 syndrome*)
51. TI sacral* n2 (pain* or facet* or nerve root* or osteoarthr* or radicul* or stenos* or spondylo* or zygapophys* or injur* or discomfort* or dysfunction* or sore* or herniat*) or AB sacral* n2 (pain* or facet* or nerve root* or osteoarthr* or radicul* or stenos* or spondylo* or zygapophys* or injur* or discomfort* or dysfunction* or sore* or herniat*)
52. TI sacro* n2 (pain* or facet* or nerve* n2 root* or osteoarthr* or radicul* or stenos* or spondylo* or zygapohys* or injur* or trauma* or discomfort* or dysfunction* or sore* or herniat*) or AB sacro* n2 (pain* or facet* or nerve* n2 root* or osteoarthr* or radicul* or stenos* or spondylo* or zygapohys* or injur* or trauma* or discomfort* or dysfunction* or sore* or herniat*)
53. TI (sacrococcygeal n2 pain*) or AB (sacrococcygeal n2 pain*)
54. TI "SI joint" n2 (pain* or facet* or nerve root* or osteoarthr* or radicul* or stenos* or spondylo* or zygapophys* or injur* or discomfort* or dysfunction* or sore* or herniat*) or AB "SI joint" n2 (pain* or facet* or nerve root* or osteoarthr* or radicul* or stenos* or spondylo* or zygapophys* or injur* or discomfort* or dysfunction* or sore* or herniat*)
55. TI sciatic* or AB sciatic*
56. TI stenos* n2 (spine* or spinal* or vertebral*) or AB stenos* n2 (spine* or spinal* or vertebral*)
57. TI osteoarthr* n2 (spine* or spinal*) or AB osteoarthr* n2 (spine* or spinal*)
58. TI condition* n2 (spine* or spinal*) or AB condition* n2 (spine* or spinal*)
59. TI disease* n2 (spine* or spinal*) or AB disease* n2 (spine* or spinal*)
60. TI disabilit* n2 (spine* or spinal*) or AB disabilit* n2 (spine* or spinal*)
61. TI disorder* n2 (spine* or spinal*) or AB disorder* n2 (spine* or spinal*)
62. TI degenerat* n2 (spine* or spinal*) or AB degenerat* n2 (spine* or spinal*)
63. TI pain* n2 (spine* or spinal*) or AB pain* n2 (spine* or spinal*)
64. TI spondylo* or AB spondylo*
65. TI tailbone n2 pain* or AB tailbone n2 pain*
66. TI vertebrogenic* n2 pain* or AB vertebrogenic* n2 pain*
67. TI (polyradicul* or poly-radicul*) or AB (polyradicul* or poly-radicul*)
68. TI neuropath* n2 (lumbar* or lumbo* or sacral* or sacro* or low back or lower back or low-back* or lower-back* or spine* or spinal* or L1 or L2 or L3 or L4 or L5) or AB neuropath* n2 (lumbar* or lumbo* or sacral* or sacro* or low back or lower back or low-back* or lower-back* or spine* or spinal* or L1 or L2 or L3 or L4 or L5)
69. TI radiculopath* n2 (lumbar* or lumbo* or sacral* or sacro* or low back or lower back or low-back* or lower-back* or spine* or spinal* or L1 or L2 or L3 or L4 or L5) or AB radiculopath* n2 (lumbar* or lumbo* or sacral* or sacro* or low back or lower back or low-back* or lower-back* or spine* or spinal* or L1 or L2 or L3 or L4 or L5)
70. TI radiating* n2 (lumbar* or lumbo* or sacral* or sacro* or low back or lower back or low-back* or lower-back* or spine* or spinal* or L1 or L2 or L3 or L4 or L5) or AB radiating* n2 (lumbar* or lumbo* or sacral* or sacro* or low back or lower back or low-back* or lower-back* or spine* or spinal* or L1 or L2 or L3 or L4 or L5)
71. TI radicular* n2 (lumbar* or lumbo* or sacral* or sacro* or low back or lower back or low-back* or lower-back* or spine* or spinal* or L1 or L2 or L3 or L4 or L5) or AB radicular* n2 (lumbar* or lumbo* or sacral* or sacro* or low back or lower back or low-back* or lower-back* or spine* or spinal* or L1 or L2 or L3 or L4 or L5)
72. TI lumborum* or longissimus or erector n2 spin* or AB lumborum* or longissimus or erector n2 spin*
73. TI synovial* n2 cyst* or AB synovial* n2 cyst*
74. TI thoracolumbar* n2 (pain* or facet* or nerve* n2 root* or osteoarthr* or radicul* or stenos* or spondylo* or zygapohys* or injur* or trauma* or discomfort* or dysfunction* or sore* or herniat*) or AB thoracolumbar* n2 (pain* or facet* or nerve* n2 root* or osteoarthr* or radicul* or stenos* or spondylo* or zygapohys* or injur* or trauma* or discomfort* or dysfunction* or sore* or herniat*)
75. TI thoraco-lumbar* n2 (pain* or facet* or nerve* n2 root* or osteoarthr* or radicul* or stenos* or spondylo* or zygapohys* or injur* or trauma* or discomfort* or dysfunction* or sore* or herniat*) or AB thoraco-lumbar* n2 (pain* or facet* or nerve* n2 root* or osteoarthr* or radicul* or stenos* or spondylo* or zygapohys* or injur* or trauma* or discomfort* or dysfunction* or sore* or herniat*)
76. TI curvatur* n2 (spine* or spinal*) or AB curvatur* n2 (spine* or spinal*)
77. OR /150-225

3. Military

1. MH Military Facilities
2. MH Military Personnel
3. MH Military Medicine
4. MH United states Department of Defense
5. MH United states Department of Veterans Affairs
6. MH Warfare
7. TI armed* n2 force* or AB armed n2 force* or TI Canadian force* or AB Canadian force* or TI American force* or AB American force*
8. TI (army* or armies*) or AB (army* or armies*)
9. TI militar* or AB militar*
10. TI active dut* or AB active dut*
11. TI air force* or AB air force*
12. TI (navy* or navies*) or AB (navy* or navies*)
13. TI marine* or AB marine*
14. TI sailor* or AB sailor*
15. TI soldier* or AB soldier*
16. TI TI fighter pilot* or AB fighter pilot*
17. TI (service* n2 member*) or AB (service* n2 member*)
18. TI "Department of Defense" or AB "Department of Defense"
19. TI "Department of Defence" or AB "Department of Defence"
20. TI Defence Department* or AB Defence Department*
21. TI Defense Department* or AB Defense Department*
22. TI (war or wars or warfare*) or AB (war or wars or warfare*)
23. TI coast guard* or AB coast guard*
24. TI national guard or AB national guard
25. TI conscript* or AB conscript*
26. TI force* n2 (defence or defense) or AB force* n2 (defence or defense)
27. TI (limited-duty or limited duty) or AB (limited-duty or limited duty)
28. TI batallion* or AB batallion*
29. TI marching or AB marching
30. TI submarin* or AB submarin*
31. TI veteran* or AB veteran*
32. TI department* n2 (defence or defense) or AB department* n2 (defence or defense)
33. OR/227 – 258
34. 149 AND 226 AND 259
35. Limit 260 NOT PT (comment or editorial or letter or clinical conference or guideline or practice guideline or case reports)
36. Limit 261 English Language
